# Growth dynamics of galls and chemical defence response of *Pinus thunbergii* Parl. to the pine needle gall midge, *Thecodiplosis japonensis* Uchida & Inouye (Diptera: Cecidomyiidae)

**DOI:** 10.1038/s41598-020-69231-4

**Published:** 2020-07-23

**Authors:** Yukun Qi, Chunhua Duan, Lili Ren, Haiwei Wu

**Affiliations:** 1 Shandong Academy of Forestry, Wenhuadong Road, Jinan, Shandong 250014 People’s Republic of China; 2 Beijing Forestry University, Beijing, 100083 People’s Republic of China

**Keywords:** Forestry, Invasive species

## Abstract

The pine needle gall midge, *Thecodiplosis japonensis* Uchida et Inouye, is a newly invasive pest in China that mainly harms *Pinus thunbergii* and *P. densiflora*. The occurrence and damage caused by *T. japonensis* in pure stands of *P. thunbergii* were investigated, and the needle growth and needle compound content were measured. Based on the above steps, the growth dynamics of galls and chemical defense response of *P. thunbergii* to attack by the gall midge were revealed. The results showed that the adults of *T. japonensis* in Qingdao city, China, emerged from the end of May to late July, with a peak in mid-June. Needles of *P. thunbergii* began to differentiate in late June and stopped growing in mid-September. The length of infested needles was 60.17% less than that of healthy needles. On average, there were 9 ± 4 larvae in each gall, 22 at most and 1 at least. The number of larvae within a gall had no significant effect on the size of the gall or larvae. Compared with that in the ungalled tissues, the content of amino acids in the galled pine needle tissues increased by 40.83%, while the content of total polyphenols, tannins, carotenoids, total triterpenes, total alkaloids and other secondary substances decreased to varying degrees, which was favourable for the growth and development of the *T. japonensis* larvae.

## Introduction

There are approximately one million named insects and more than 500,000 plants on Earth, accounting for more than half of the total global biodiversity^77^. During the long-term process of natural selection, plants and insects are closely related in regard to nutrition, reproduction, protection, defence and diffusion. Plants provide food and habitat for insects, but insects also provide many benefits to plants, such as seed dispersal and pollination^37^. There are many ways by which insects obtain nutrition from plants. Some insects, such as beetles, moths, and wasps, directly eat the leaves, stems and roots of plants, which affects the absorption of nutrients and the performance of photosynthesis by plants^26,54,74^. Some suck the sap from plant tissue, such as aphids, red spiders, and whiteflies^13,19,28^, which causes the loss of green colouration, the deformity of plant tissue and plant death. In general, host plants respond positively to damage caused by pests. For instance, almost all secondary metabolites, including nitrogenous compounds (such as alkaloids and non-protein amino acids), terpenes (such as monoterpenes and diterpenoids), and phenols (such as monophenols and flavonoids), may be produced in large quantities in plant tissues due to insect attack^2,9,16,20,34,41,73^.

The insects that can induce plants to produce galls are called gall-forming insects and represent a large group of phytophagous insects. Galls on host plants vary morphologically among different insects^27^, these morphological differences are considered to represent the adaptability of the gall-forming insects to the environment^5,27,33^. Six hypotheses have been proposed to explain this phenomenon, including the nonadaptive hypothesis, plant protection hypothesis, mutual benefit hypothesis, nutrition hypothesis, microenvironment hypothesis, and enemy hypotheses^36^. According to incomplete statistics, gall-forming insects mainly belong to seven orders and 20 families, and estimates of their global richness range from 21,000 to 211,000 species, with an average of 132,930 species^6,10,40^. Because gall-forming insects are often associated with a single host plant species, gall-forming insects and their plant hosts represent the best model for exploring the relationships between insects and plants^29,33,50^.

**Figure 1:**
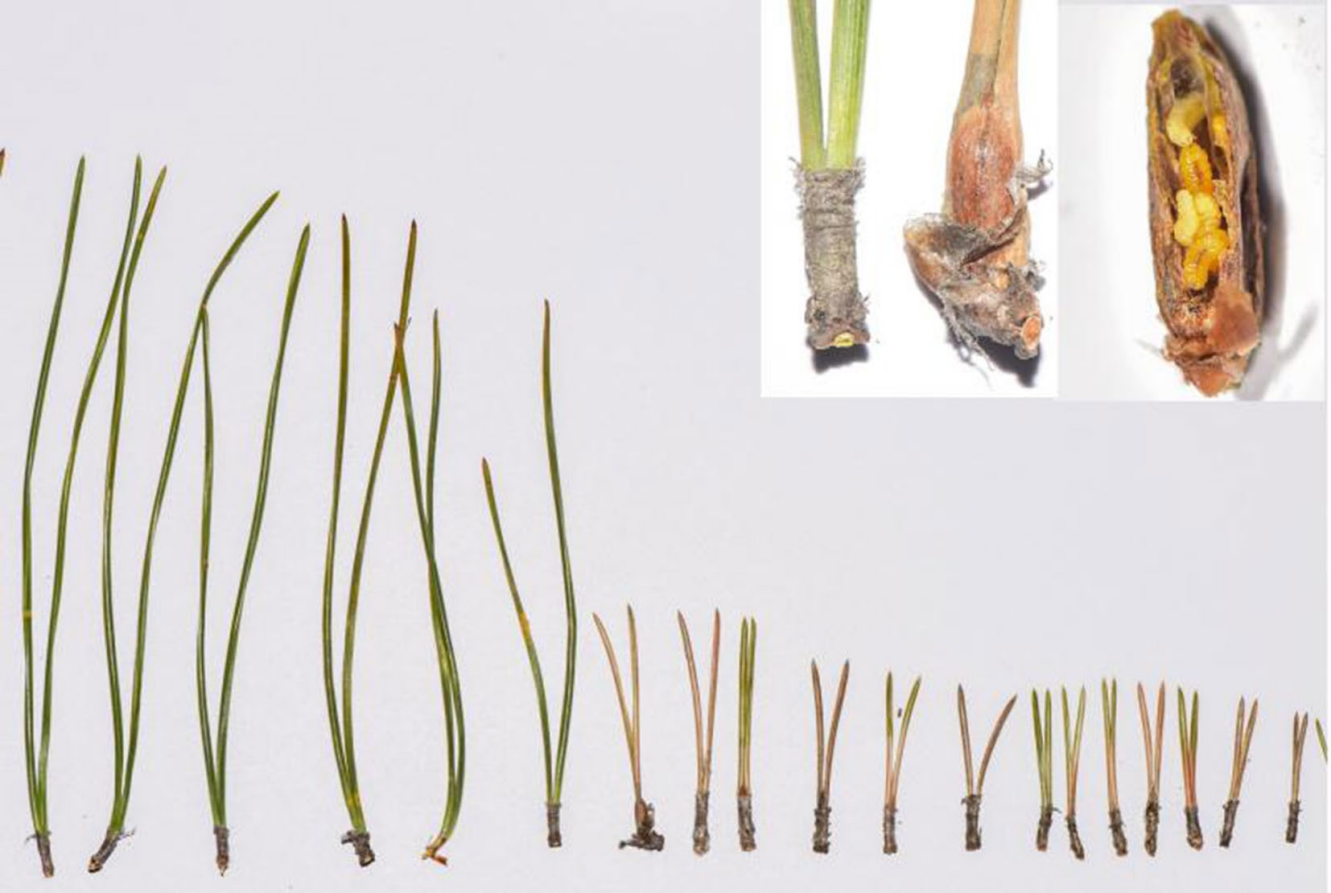
Galls of *T. japonensis* and larvae in galls in needle pairs of *Pinus thunbergii.* Long needle pairs were unharmed, and short needle pairs were attacked.

Cecidomyiidae is now consisting of 6,590 species, but only 75% of them are gall inducers^11^. The pine needle gall midge (*Thecodiplosis japonensis*) originated in Japan and was first recorded as a pest in Seoul and Chonnam Province in Korea in 1929. It has been recorded across the whole territory of Korea since 1960 and has become one of the most serious pests in South Korean pine forests^18^. *T. japonensis* occurred one generation a year. Larvae hibernate in the soil, where they pupate in early May. Adults emerge from the litter from the end of May until July. After mating, females search for a suitable host plant (*Pinus densiflora* or *P. thunbergii*) and lay eggs on needle pairs of current-year shoots in the vicinity of where they emerge from the soil. Larvae hatch from their eggs after approximately 1 week. The newly hatched larvae move to the base of the needles, where they begin to form galls^31^. In 2016, the pest was first found in Huangdao, China (Fig. 1), and showed a trend of gradual expansion.

In most cases, there are more than two *T. japonensis* larvae per gall. The gregariousness of the larvae within a gall does not cause the death of larvae due to the restriction of food and space. They adapt to the gall environment by undergoing a reduction in body size and fertility^45^. It has been shown that phenolic compounds in needles and the resin secreted by host plants can kill the hatched larvae^43,44,47,48^. Based on these results, we speculate that there are some very interesting problems that need to be solved: (1) is there a positive correlation between the number of larvae in galls and the size of gall? Will the larger number induce larger galls? (2) How did the host plant resist the damage of the pine needle gall midge proactively, are there physiological and morphological changes in plant tissue? To address these questions, we investigated the characteristics of the occurrence of pine needle gall midges and evaluated the growth dynamics of pine needles and the content of compounds in different tissues in the needles of infested hosts.

## Study site

The midge population was surveyed in a pure stand of *P. thunbergii* in Huangdao district, Qingdao city, Shandong Province (35° 58′ 14″ N, 120° 11′ 28″ E). The mean tree height and the mean diameter at breast height were 6.5 m and 12 cm, respectively. The main undergrowth vegetation includes *Bidens pilosa*, *Imperata cylindrica* and *Humulus scandens*. The annual average temperature is 12.5 ℃, and the annual average rainfall is 750 mm.

## Materials and methods

### Growth of needle pairs

Ten current-year shoots were randomly marked in the forest stand with a black marker pen, and five needle pairs on each current year shoot were randomly marked and numbered. The length of each needle pair was measured with a Vernier caliper every 10 days from the first ten days of May to the middle ten days of November 2019. After the last observation, all the observed needle pairs were taken back to the laboratory to count the number of larvae per gall.

### Gall growth

Ten current-year shoots of black pine were randomly sampled every 5–8 days from July to September 2019. Five infested needle pairs were randomly selected from each shoot, and the length and width of the galled and ungalled tissues were measured with a Vernier caliper. The current-year shoots were collected four times per month, and more than 30 galls were treated each time.

### Adult emergence

The number of emerging adults was surveyed by using 30 emergence traps. An emergence trap was composed of an opaque vinyl chloride pipe 10.5 cm in diameter and 15 cm in height and a transparent plate coated with a viscous substance^47^. The emerging adults were counted a dissection microscope (Leica EZ4HD) at intervals of 3–5 days during the period from May 20 to July 30, 2019.

### Larvae in galls

Thirty current-year shoots of black pine were randomly sampled every 5–8 days from early October to early November 2019. Ten infested needle pairs were randomly selected from each shoot, and the length and width of the ungalled and galled tissue were measured. Then, each gall was dissected longitudinally with a scalpel to count the number of larvae (the dissected gall was placed in a Petri dish containing pure water (φ = 5 cm, water depth = approximately 2 mm), and the pine needle gall midge larvae climbed out automatically). No fewer than 300 galls were treated. The galls in which larvae were found to be parasitic were excluded.

### Relationship between population number and body type of the larvae in galls

The larvae that crawled out of galls with populations of 5, 6, 7, 8 or 9 larvae were frozen in the refrigerator for 2–3 min, and then the body length and body width of each larva were measured under an anatomical microscope. No fewer than three galls were treated in the different groups.

### Determination of coniferous compounds

Fifty current-year shoots of black pine were randomly sampled. Thirty infested and healthy needle pairs were selected from each shoot and then immediately placed in an ice bottle in the field. After the samples were transported to the laboratory, they were stored in a refrigerator at − 20 ℃ for future use.

The amino acid content was determined according to the ethanol extraction method and hydrochloric acid hydrolysis method^25^. The total phenol content was determined with the Folin phenol method^72^, and the tannin content was determined with sodium tungstate-phosphomolybdic acid colorimetry^7^. The total chlorophyll, carotenoid and total triterpene content was evaluated with UV spectrophotometry^55,71^. The total alkaloid content was assessed with acid dye colorimetry^25^, and the organic acid content was determined according to the high-performance liquid chromatography method^32^.

There were four treatments for each index: galled tissue of the attacked needle pairs (T1), ungalled tissue of the attacked needle pairs (T2), base tissue of the healthy needle pairs (CK1, the control of T1) and upper tissue of the healthy needle pairs (CK2, the control of T2).

### Data analysis

Since the needle galls on *P. thunbergii* are approximately cylindrical, the volume (*V*) and the relative volume (*RV*) of the galls can be calculated using the following formula:

$$V = 3.14 \times GD \div 2^{2} \times L$$

$$RV = 3.14 \times \left( {GD \div 2^{2} - HD \div 2^{2} } \right) \times GL$$

where GD represents gall width, GL represents gall length, and HD is the width of ungalled tissue.

One-way ANOVA was used to test the effects of the growth period on the length, width, volume, and relative volume of the galls and the number of larvae in the galls on the length, width, volume, relative volume of the galls and larval size. The ARTAN function was used for data conversion for the length of the galls, and the logarithmic function LG (10) and SQRT function were used for data conversion for the volume and relative volume of the galls to ensure homogeneity of variance. Statistical analyses were performed using SPSS 18.0.

## Results and analysis

### Response of pine needle growth

#### Growth dynamics of the pine needles

**Figure 2:**
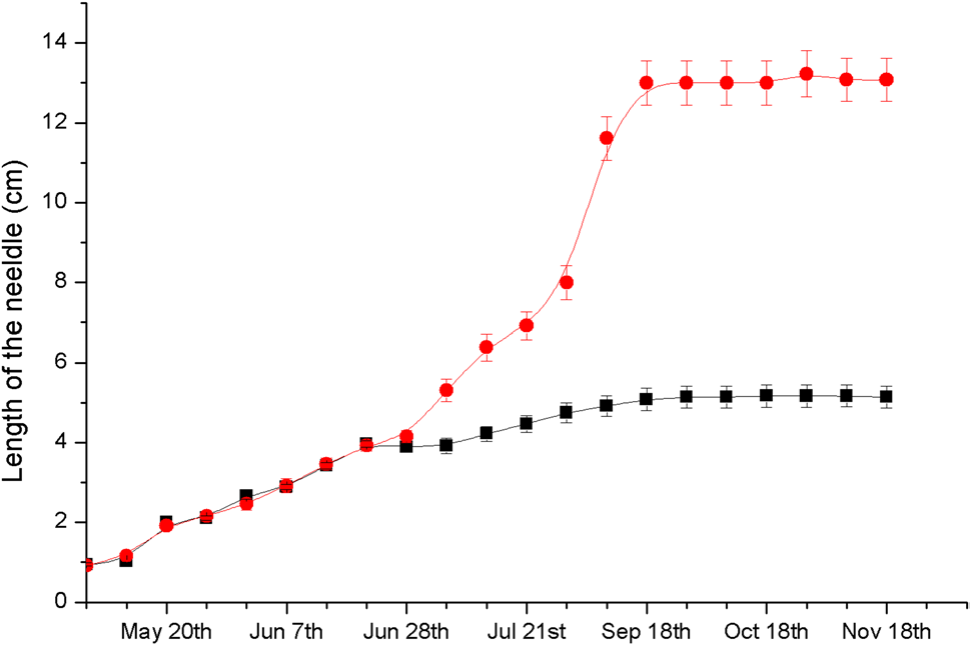
Changes in pine needle growth in 2019. Solid circles indicate the mean length of the attacked needle pairs of *P. thunbergii*. Solid squares indicate the mean length of the healthy needle pairs of *P. thunbergii*. Error bars show the standard error of the means.

Needles of *P. thunbergii* began to differentiate in late June. Some of them maintained rapid elongation, and some grew slowly. All the needles discontinued their growth and elongation in the middle of September, and the length of the needles that grew slowly was 60.17% less than that of the others on average (Fig. 2). The anatomical results showed that there was a gall in each short needle, and more than one pine needle gall midge could be found in each gall.

#### Adult emergence dynamics

**Figure 3:**
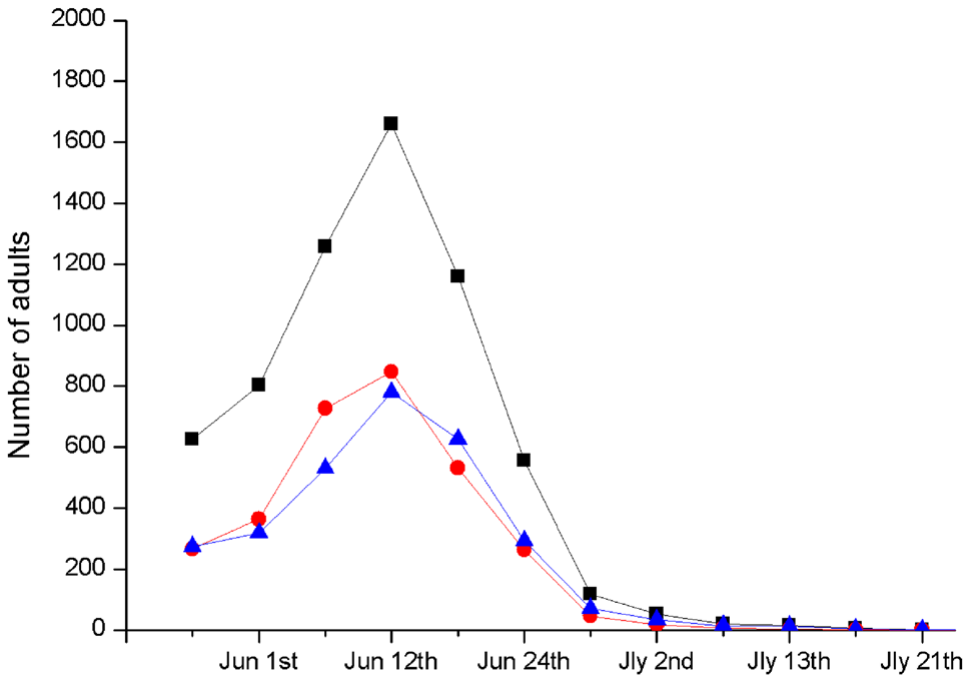
Eclosion rhythm of *T. japonensis* in 2019. Solid squares indicate the total number of adults. Solid circles indicate the number of female adults. Black triangles represent the number of male adults.

**Figure 4:**
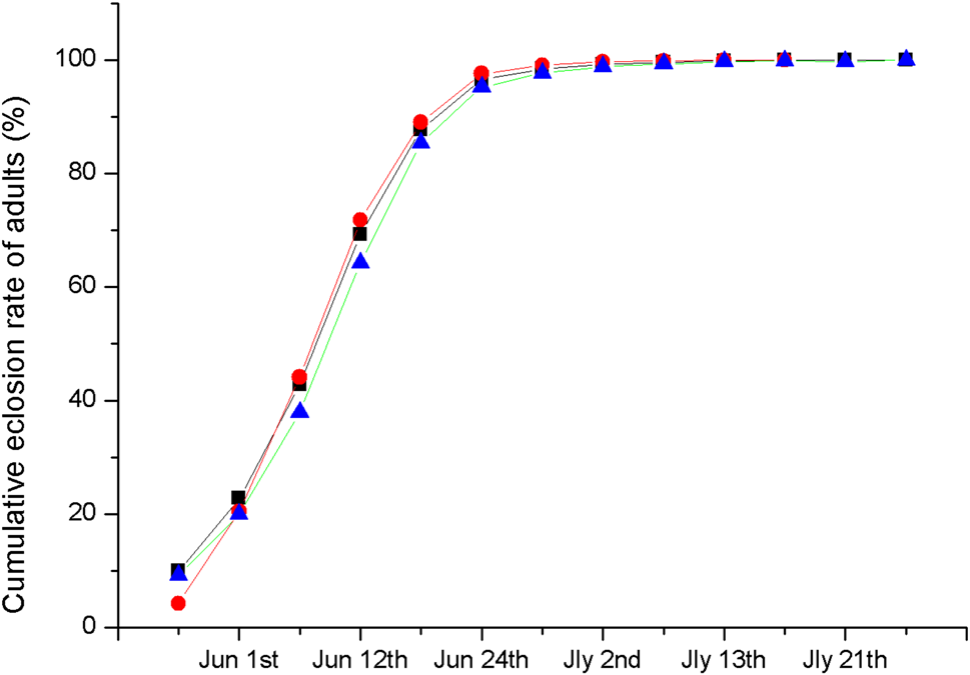
Cumulative emergence rate of *T. japonensis* in 2019. Solid squares indicate the total number of adults. Solid circles indicate the number of female adults. Black triangles represent the number of male adults.

In the field, the adults of *T. japonensis* emerged from the end of May to late July, with a peak in mid-June (Figs. 3, 4). The number of adults that emerged before June 20 accounted for 87.72% of the annual emergence number, and this percentage reached 98.45% on June 28. Both the female and male adults had the same emergence rhythms as the total population. However, there were more female adults than male adults in the early stages, with a sex ratio of 1.14:1 (female to male). In the later period, this ratio was 0.60:1.

#### Gall growth

In July, August and September, the average gall lengths were 7.68 mm, 9.75 mm and 11.01 mm, respectively, and the widths were 1.51 mm, 1.84 mm and 1.95 mm, respectively. In comparison with the ungalled tissues, the width of the galled tissues increased by 0.25 mm, 0.47 mm and 0.51 mm, respectively (Table 1). The statistical results showed that the length, width, relative width, volume and relative volume of the galls significantly differed among the months (gall length: *F*_(2,477)_ = 161.910, *P* = 0.000, gall width: *F*_(2,477)_ = 73.146, *P* = 0.000; gall relative width: *F*_(2,413)_ = 22.735, *P* = 0.000; gall volume: *F*_(2,413)_ = 91.920, *P* = 0.000; relative gall volume: *F*_(2,413)_ = 56.006, *P* = 0.000), which indicates that the galls also increased in size with the growth of the needles.

**Table 1:** Mean growth index values of the galls on attacked needle pairs from July to September in 2019.

Month	Gall length (mm)	Gall width (mm)	Relative width of gall* (mm)	Gall volume (mm^3^)	Relative volume of gall (mm^3^)
7	7.68c	1.51c	0.25c	16.23c	4.74c
8	9.75b	1.84b	0.47ab	28.00b	12.25b
9	11.01a	1.95a	0.51a	34.16a	15.79a

*${\text{Relative width of gall}} = {\text{width of galled tissues}} - {\text{width of ungalled tissue}}$. Letters following the numbers indicate the significance of the means.

### Relationship between the number of larvae and galls

#### Quantitative characteristics of T. japonensis larvae in galls

**Figure 5:**
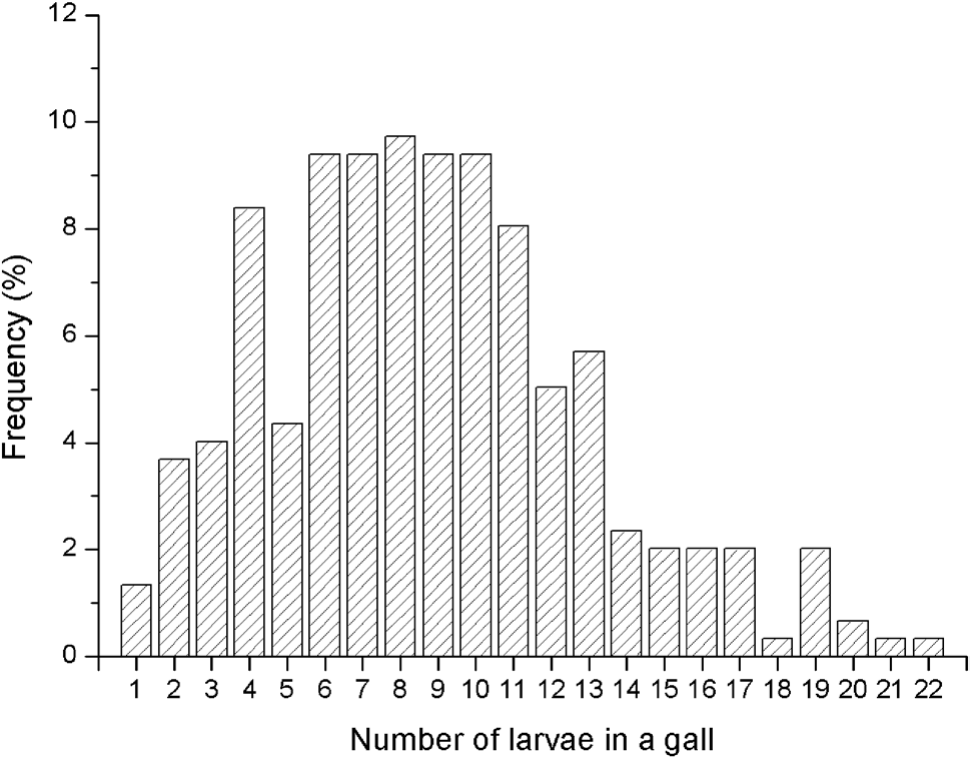
Frequency distribution of the number of larvae in a gall. Arrows indicate the mean number of larvae per gall.

The larvae of *T. japonensis* usually formed a gall at the base of the needle pair on the current year shoot, but occasionally they formed it in the middle of the needle pair. On average, there were 9 ± 4 larvae in each gall, 22 at most and 1 at least. The number of larvae in the galls was mostly 6–11, accounting for 55.37% of the total (Fig. 5).

#### Relationship between the number of T. japonensis and gall size

Analysis of variance showed that the number of larvae within a gall had no significant effect on the length, width, volume, or relative volume of the gall (gall length: *F*_(17,275)_ = 1.176, *P* = 0.283; gall width: *F*_(17,275)_ = 0.467, *P* = 0.966; gall volume: *F*_(17,275)_ = 0.458, *P* = 0.969; relative gall volume: *F*_(17,275)_ = 0.672, *P* = 0.829). In other words, although the number of larvae damaging the base of the needles differed, the gall-forming effects were almost the same.

#### Differences in individual size

**Figure 6:**
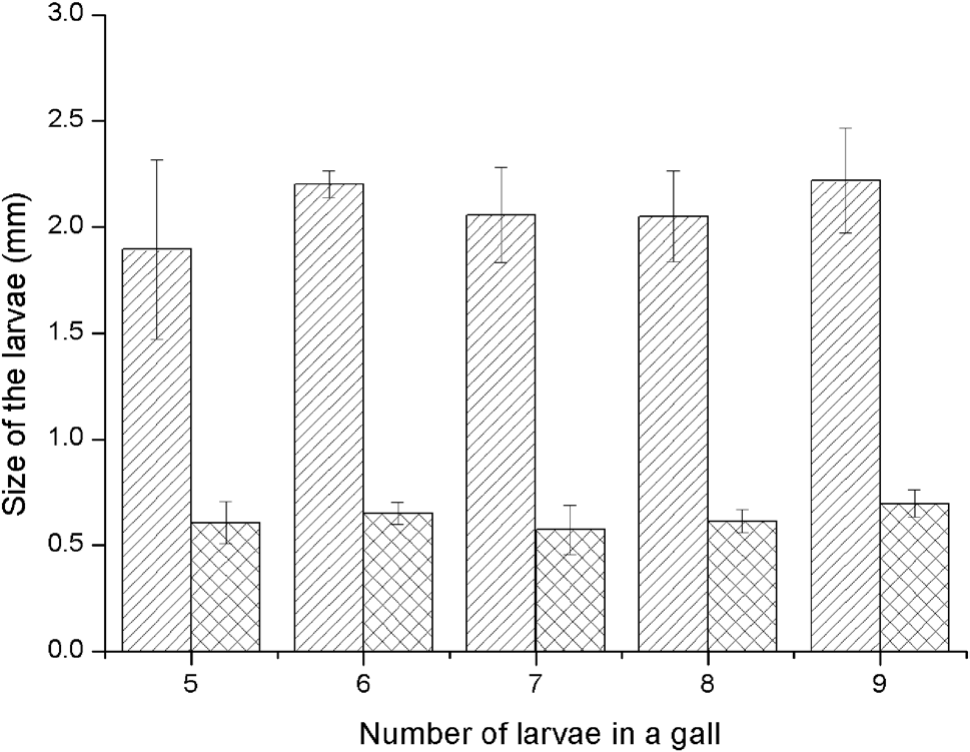
Body size of mature larvae of *T. japonensis* in a gall. Slash-filled columns represent the mean body length. Grid-filled columns indicate the mean body width. Error bars show the standard error of the means.

The mean body lengths of mature *T. japonensis* larvae were 1.90 mm, 2.20 mm, 2.06 mm, 2.05 mm and 2.22 mm, respectively, for the different numbers (5, 6, 7, 8 and 9) larvae within a gall, and the corresponding mean body widths were 0.61 mm, 0.65 mm, 0.57 mm, 0.61 mm and 0.70 mm, respectively (Fig. 6). The statistical results show that the number of *T. japonensis* within a gall had no significant effect on the size of the individuals (average body length: *F*_(4,13)_ = 1.074, *P* = 0.409,average body width: *F*_(4,13)_ = 1.355, *P* = 0.302).

### Chemical defence response of P. thunbergii

#### Primary metabolite content

**Figure 7:**
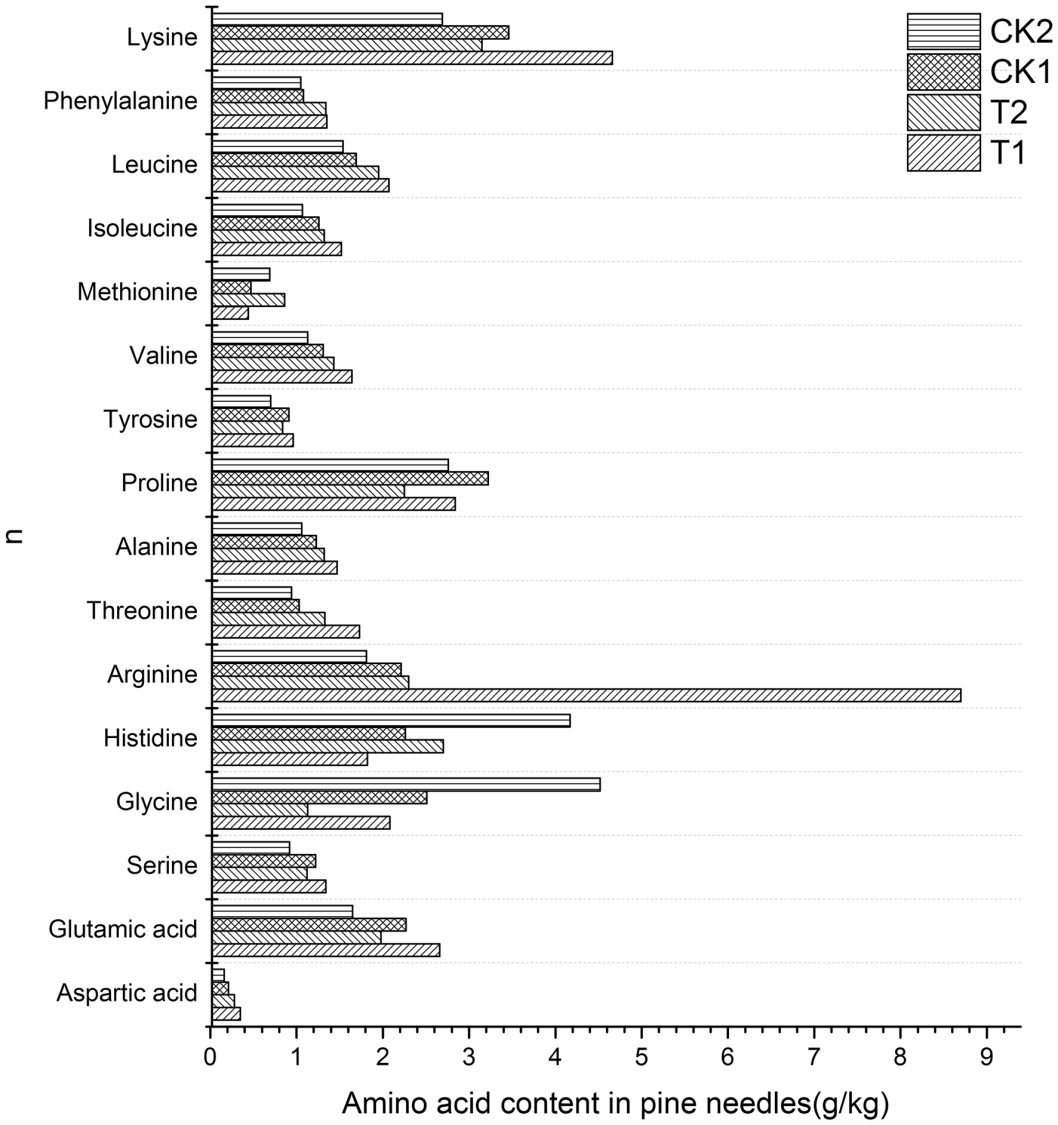
Content of different amino acids in pine needles. Forward slash-filled columns represent the content of different amino acids in galled tissue of the attacked needle pairs (T1); backslash-filled columns indicate the content of different amino acids in ungalled tissue of the attacked needle pairs (T2); grid-filled columns represent the content of different amino acids in the base tissue of the healthy needle pairs (CK1, the control of T1); horizontally filled columns indicate the content of different amino acids in the upper tissue of the healthy needle pairs (CK2, the control of T2).

The results showed that the content of amino acids in the galled tissue of attacked needle pairs was significantly higher than that in the ungalled tissue and the control, increasing by 40.83% and 35.27%, respectively. The contents of 12 amino acids, including lysine, phenylalanine, leucine, isoleucine, valine, tyrosine, alanine, threonine, arginine, serine, glutamic acid and aspartic acid, all increased to varying degrees. In particular, the content of arginine in galled tissue increased by 293.67% compared with that in the control and by 278.26% compared with that in ungalled tissue, followed by threonine and aspartic acid, which increased by more than 65% in all cases. In contrast, the contents of methionine, proline, histidine, and glycine in the galled tissue of the attacked needle pairs decreased compared with that in the control, among which the largest decrease was observed for histidine, which decreased by 19.47% (Fig. 7).

**Figure 8:**
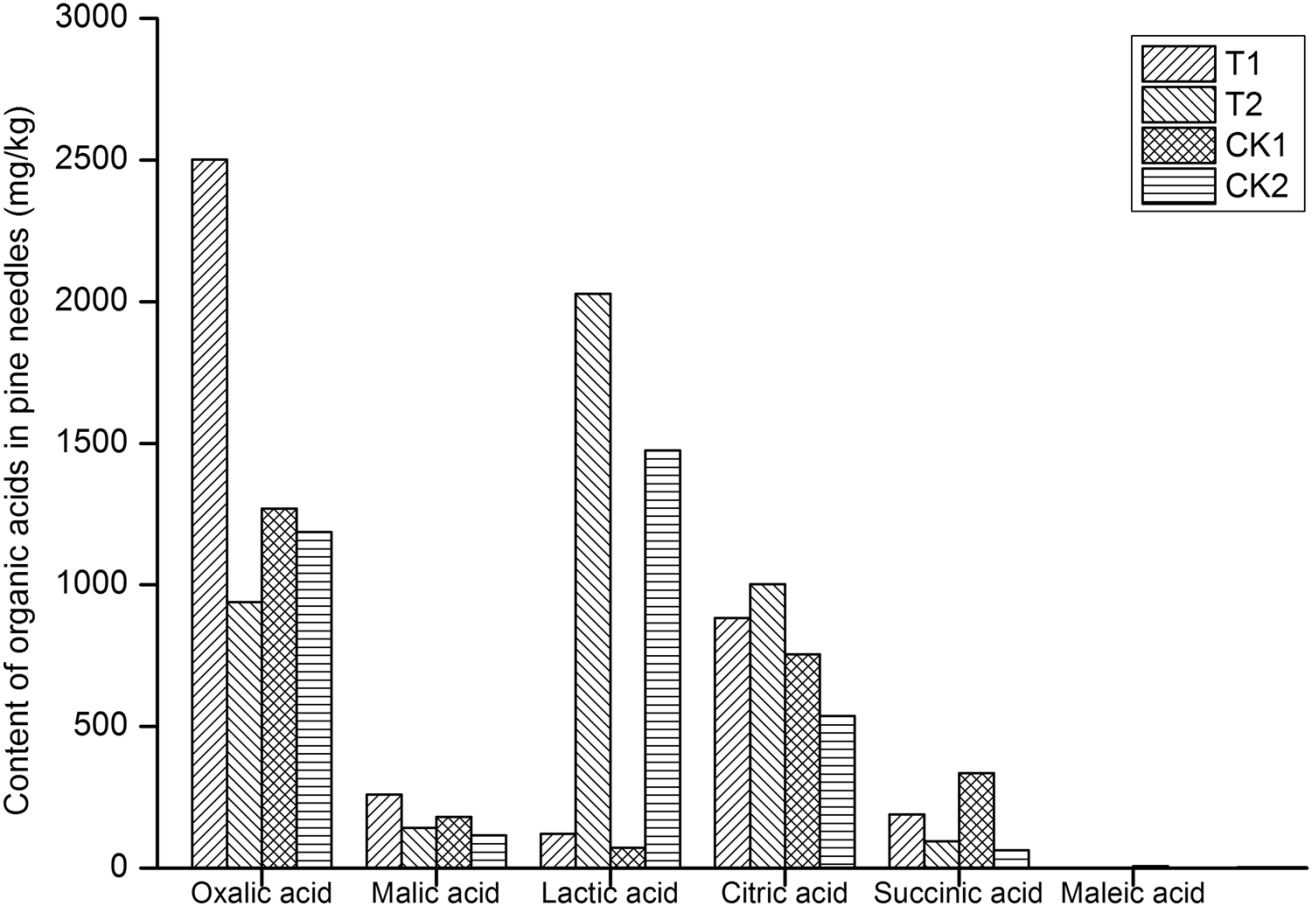
Content of different organic acids in pine needles. Forward slash-filled columns represent the content of different organic acids in galled tissue of the attacked needle pairs (T1); backslash-filled columns indicate the content of different organic acids in ungalled tissue of the attacked needle pairs (T2); grid-filled columns represent the content of different organic acids in the base tissue of the healthy needle pairs (CK1, the control of T1); horizontally filled columns indicate the content of different organic acids in the upper tissue of the healthy needle pairs (CK2, the control of T2).

Compared with the contents in ungalled tissues, the contents of oxalic acid, malic acid, succinic acid and fumaric acid increased by 166.05%, 81.76%, 99.28% and 0.95%, respectively, in the galled tissues of attacked needle pairs, while the contents of lactic acid, citric acid and maleic acid decreased by 94.00%, 11.81% and 3.11%, respectively. Compared with those in the control (CK1), the contents of oxalic acid, malic acid, lactic acid, citric acid and fumaric acid increased by 96.88%, 43.53%, 69.83%, 16.98%, and 101.42%, respectively, in the galled tissues of attacked needle pairs, while those of succinic acid and maleic acid decreased by 43.35% and 65.51%, respectively (Fig. 8).

#### Secondary metabolite content

**Figure 9:**
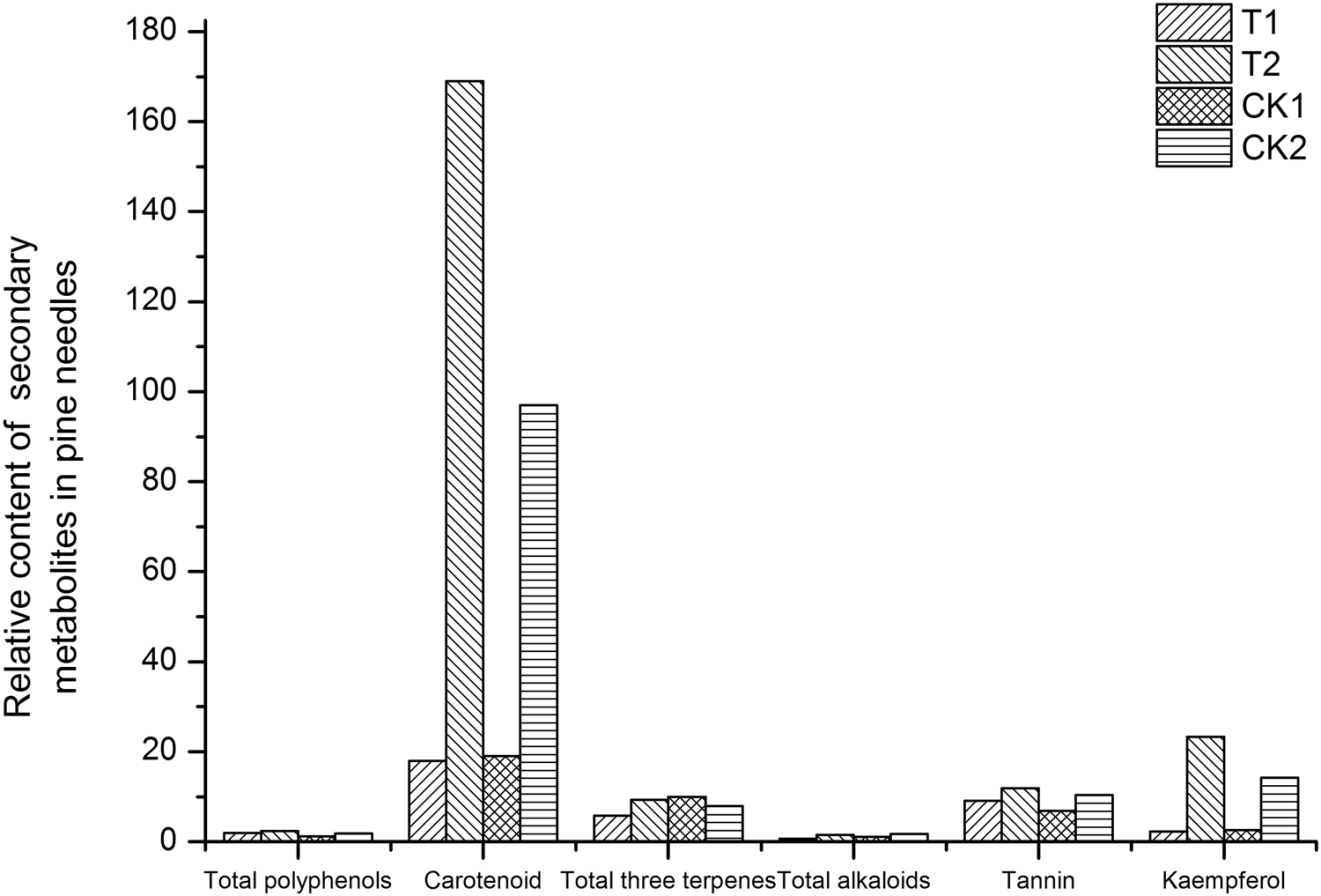
Relative content of different secondary metabolites in pine needles. Forward slash-filled columns represent the content of different secondary metabolites in galled tissue of the attacked needle pairs (T1); backslash-filled columns indicate the content of different secondary metabolites in ungalled tissue of the attacked needle pairs (T2); grid-filled columns represent the content of different secondary metabolites in the base tissue of the healthy needle pairs (CK1, the control of T1); horizontally filled columns indicate the content of different secondary metabolites in the upper tissue of the healthy needle pairs (CK2, the control of T2).

The contents of total polyphenols and tannins in the galled tissue of attacked needle pairs increased by 58.33% and 32.29%, respectively, in comparison to those in the control, while the contents of carotenoids, total triterpenoids, total alkaloids and kaempferol decreased by 5.26%, 42.00%, 36.36% and 9.77%, respectively (Fig. 9). Compared with those in the ungalled tissue, the contents of the six secondary metabolites tested all decreased to different degrees in the galled tissue.

## Discussion

### Damage to P. thunbergii by T. japonensis

Similar to the findings of^31^ in Korea, adults of *T. japonensis* in the field in Qindao city, China, emerged from the end of May to late July, with a peak in mid-June. Only one gall formed by the larvae of *T. japonensis* was usually found at the base of the needle pair on the current year shoot, and few galls were formed in the middle parts. The number of larvae per gall found in the present study (mostly 6–11, with an average of 9 ± 4) was higher than that found by Soné^47^ in a young stand of *P. thunbergii* damaged by pine needle gall midges for six consecutive years from 1976 to 1981. This author^47^ also reported that the mean body length of the mature larvae gradually decreased as the number of larvae per gall increased from 1 to 12. This finding was also in disagreement with our results, as no significant differences were found in the body size of the larvae in relation with the number of larvae in the galls, at least for the range of 5–9 larvae considered in our study. Further research should be performed to evaluate this aspect out of that range.

### Growth dynamics of P. thunbergii needles

As found in other pine trees damaged by gall midges^42^, the attacked pine needles of *P. thunbergii* are significantly shorter than the healthy needles, although the needle shortening rate varies among different hosts. According to the observations of Lee and Sung^17^, the shortening rates of *P. densiflora* and *P. rigida* needles were 48.1% and 37.45%, respectively. Our results show that the shortening rate of the attacked needle pairs of *P. thunbergii* was approximately 60%, which could suggest that *P. thunbergii* might be more sensitive to the effects of the galling insect than *P. densiflora* and *P. rigida*. In addition, the growth synchronicity of attacked and unattacked needle pairs differs among different hosts. According to the report by Chung et al.^4^, needle pairs of *P. densiflora* attacked by *T. japonensis* discontinued their growth and elongation in early July, while the healthy needles could grow and elongate linearly from early May to the middle of August. Our investigation results show that the needles of *P. thunbergii* began to differentiate in late June. The undamaged pine needles maintained rapid elongation, and the attacked needles grew slowly. Both needle types discontinued their growth and elongation in middle September. It can be assumed that the gall may drain water and carbon towards the nutrition of the larvae in detriment of the development of the needles, and this may be a positive response strategy used by pines to deal with the damage caused by gall midges, that is, limiting resources towards the galls. The shorter the needle pairs, the smaller should the galls be.

### Restrictive effect of galls on the number of larvae

There are four main views regarding the factors affecting the growth and development of galls. ① As indicated by Weiss et al.^59^ “galls are phenotypic entities that develop under the influence of both plant and insect genotypes”. The size of the galls is the result of natural selection. Depending on the balance among the causes of mortality for a given population, the net selective pressure may favour either an increase or a decrease in gall size. ② The size of galls is closely related to the number of gall-forming insects. A larger population results in a larger gall size^64,76^. ③ The development of galls has nothing to do with the number of gall-forming insects. Gall-forming insects only have a priming effect during gall formation^52,63,65,66^. ④ The size of galls depends on the activity of sucrose-degrading enzymes^53,62^. Our observations support the third view mentioned above. Galls caused by *T. japonensis* did not vary in size and the number larvae inside them may be either big (22) or small (1). Additionally, the size of the larvae did not vary either (Fig. 6).

### Chemical defence response of black pine

Upon attack by insects, plants emit over 200 different volatile organic compounds^8^, and some of them are secondary metabolites^35^. Among plant secondary metabolites, plant polyphenols (including tannins, flavonoids, etc.), alkaloids and triterpenoids have been well documented to act as toxins, feeding deterrents, or oviposition deterrents to a range of insects^3,56,58,67,75^. Plant carotenoids have been found to be insect feeding deterrents and have fundamental roles as antioxidants in plant growth and development^39,60^. Our results showed that the contents of total polyphenols, total triterpenes, tannins and carotenoids in attacked needle pairs of *P. thunbergii* are higher than those in control (Fig. 9), which may be a positive response strategy used by pines to deal with the damage caused by gall midges.

Because galls result from a change in the plant development process induced by insects^49,51^, the chemical defence strategy of host plants against gall-forming insects is often regulated by the gall-forming insects themselves. For example, gall-forming aphids can induce plants to increase the content of compounds such as tannic acid in galled tissue, which can improve the toxic effect on most insects and protect the gall-forming insects from being parasitized or preyed upon^12,61^. Some gall-forming insects can manipulate plant enzymes and place the secondary metabolites that are harmful to themselves in the outermost layer of the gall, while the inner layer surrounding the larvae is not toxic,this process can reduce damage and promote their growth^1^. In contrast, the contents of tannins and flavonoids in galls induced by *Dryocosmus kuriphilus* were significantly lower than those in the branches of host plants^54^, and the content of malondialdehyde in galled tissue induced by thrips was lower than that in ungalled tissue^70^. The above phenomena have also been observed in studies on the ungalled tissues of *Neothoracaphis hangzhouensis* and *Tetraneura akinire*^14,23^. The contents of total polyphenols, tannins, carotenoids, total triterpenoids and total alkaloids in the galled tissue of *P. thunbergii* infested by *T. japonensis* were lower than those in ungalled tissue, which could reduce the chemical defence of the host plants and benefit the growth and development of the *T. japonensis* larvae*.*

In addition to controlling the content of secondary metabolites in host plants, gall-forming insects can also benefit from regulating the composition and content of primary metabolites. Numerous studies have shown that the content of nutrients such as soluble sugar, soluble protein, and free amino acids in galls is higher than that in normal tissues^9,16,34^. Similarly, our findings also provide evidence for these view. The determination of the free amino acid content of the attacked needle pairs showed that the amino acid content of the galled tissues was 40.83% higher than that of the ungalled tissues. Amino acids are one of the three precursors of plant secondary metabolism. Most alkaloids are produced by amino acid metabolism, such as pyrrolidine alkaloids (nicotine) derived from ornithine, piperidine alkaloids derived from aspartic acid and lysine, indole alkaloids derived from tryptophan (quinine), isoquinoline alkaloids derived from tyrosine (berberine). The metabolism of amino acids in galls may be controlled by the pine needle gall midge. They have two benefits for maintaining the amino acid content at a high level: on the one hand, it can provide nutrition for their growth and development^15,30,80^, on the other hand, it may reduce the toxic effect of alkaloids produced in the process of amino acid metabolism. For example, Proline has the function of scavenging active oxygen, which can avoid the toxicity caused by the accumulation of active oxygen produced by plant defense reaction^21,38^.

As is known, both aspartic acid and arginine has a high nitrogen-to-carbon ratio in plants. The high content of aspartic acid in gall showes that the nitrogen transport in gall tissue is vigorous^22^. Arginine participates in almost all physiological and biochemical processes in plants, including growth, development, and stress resistance^68^. It synthesizes polyamines (PA) under the catalysis of arginine decarboxylase (ADC) and regulates cell division in plants through PA^69,78^. It can be speculated that the large amount of aspartic acid and arginine may accelerate cell division in the attacked tissue of the pine needles, which may explain the mechanism of gall formation partly.

## Conclusion

The study of the newly invasive pest *T. japonensis* in Qingdao city, China, showed that it occurred one generation a year. Adults emerged from the end of May to late July, with a peak in mid-June. The larvae of *T. japonensis* usually formed a gall at the base of the needle pair on the current year shoot, but occasionally they formed it in the middle of the needle pair. There were nine larvae in each gall on average, but ranging from 1 to 22. There was not a significant correlation between the number of larvae in galls and either the gall size or the larvae size, at least for the range of 5–9 larvae considered in our study. The length of infested needles of *P. thunbergii* was approximately 60% less than that of healthy needles. While the content of amino acids in the galled pine needle tissues increased by 40.83% in comparison with the ungalled tissues, the total amount of polyphenols, tannins, carotenoids, total triterpenes, total alkaloids and other secondary substances decreased, which was favourable for the growth and development of the *T. japonensis* larvae.
